# Building a patient-centered and interprofessional training program with patients, students and care professionals: study protocol of a participatory design and evaluation study

**DOI:** 10.1186/s12913-018-3200-0

**Published:** 2018-05-30

**Authors:** Thomas W. Vijn, Hub Wollersheim, Marjan J. Faber, Cornelia R. M. G. Fluit, Jan A. M. Kremer

**Affiliations:** 1 Scientific Center for Quality of Healthcare, Radboud Institute of Health Sciences, Radboud University Medical Center, 114, PO Box 9101, 6500 HB Nijmegen, The Netherlands; 2 Radboudumc Health Academy, Department for Research in Learning and Education, Radboud University Medical Center, 43, PO Box 9101, 6500 HB Nijmegen, The Netherlands

**Keywords:** Co-production, Participatory medicine, Patient education, Medical education, Plan-do-study-act, Educational intervention, Design and evaluation study

## Abstract

**Background:**

A common approach to enhance patient-centered care is training care professionals. Additional training of patients has been shown to significantly improve patient-centeredness of care. In this participatory design and evaluation study, patient education and medical education will be combined by co-creating a patient-centered and interprofessional training program, wherein patients, students and care professionals learn together to improve patient-centeredness of care.

**Methods:**

In the design phase, scientific literature regarding interventions and effects of student-run patient education will be synthesized in a scoping review. In addition, focus group studies will be performed on the preferences of patients, students, care professionals and education professionals regarding the structure and content of the training program. Subsequently, an intervention plan of the training program will be constructed by combining these building blocks. In the evaluation phase, patients with a chronic disease, that is rheumatoid arthritis, diabetes and hypertension, and patients with an oncologic condition, that is colonic cancer and breast cancer, will learn together with medical students, nursing students and care professionals in training program cycles of three months. Process and effect evaluation will be performed using the plan-do-study-act (PDSA) method to evaluate and optimize the training program in care practice and medical education. A modified control design will be used in PDSA-cycles to ensure that students who act as control will also benefit from participating in the program.

**Discussion:**

Our participatory design and evaluation study provides an innovative approach in designing and evaluating an intervention by involving participants in all stages of the design and evaluation process. The approach is expected to enhance the effectiveness of the training program by assessing and meeting participants’ needs and preferences. Moreover, by using fast PDSA cycles and a modified control design in evaluating the training program, the training program is expected to be efficiently and rapidly implemented into and adjusted to care practice and medical education.

## Background

Patient-centeredness, first coined by Balint in 1955 [1], is currently considered to be the core ethical imperative to guide healthcare practice, education and research. Next to the ethical perspective, patient-centeredness has been shown to improve patients’ knowledge, patients’ experiences, health service use and cost, and patients’ health and well-being [2]. A common approach to stimulate patient-centered care is training care professionals in patient-centered attitudes and skills. It has been shown, however, that patient-centeredness significantly improves if patients are also trained [3]. Therefore, patient education is increasingly applied to enhance patients’ health, for example, by improving disease knowledge, self-care, health literacy, disease behavior, and health outcomes; healthcare shifts from a provider-driven approach to shared decision making [4].

Along with patient empowerment, medical education shifts towards mature roles for students in care practice [5]. Undergraduate medical students and students of other healthcare professions are increasingly involved in care practice, for example during longitudinal internships [6, 7], in service-learning education [8–10], and in student-run clinics [11–13]. By enabling students to contribute to care services and giving them a mature role in care practice in an early stage of their educational career, students’ professional identity and attitude, team experience and skills, and ability to perform tasks are improved [14].

In addition, interprofessional education is applied to enhance quality of (future) care by improving students’ attitudes towards and perceptions of other professions in healthcare, and students’ knowledge and skills with regard to collaboration in care [15] . Even more, evaluation of interprofessional training of students by patients shows that teamwork skills and understanding of and dealing with the patients’ perspective are enhanced by bringing together patients, medical students, nursing students and other future health professionals [16].

To simultaneously facilitate training of patients in managing their illness and treatment and provide learning opportunities for students and care professionals, a patient-centered and interprofessional training program for patients, students and care professionals will be constructed at the Radboudumc and Hogeschool Arnhem en Nijmegen in Nijmegen, the Netherlands. In the training program, patients, students of different professions and care professionals will work and learn together to exchange experiences, knowledge, and skills by combining patient education, service learning, workplace learning and interprofessional education. It is hypothesized that patient-centeredness of care will be improved by facilitating patients, students of different professions and care professionals to learn together and from each other. To design and evaluate the training program in care practice and medical education, a participatory design and evaluation study will be conducted.

## Methods

The concept of design-based research and the framework of the Medical Research Council (non-departmental public body of the United Kingdom aimed at supporting science and educating scientists) for developing and implementing complex interventions in healthcare are used in this study to guide the research design. Design-based research is used in educational research to design, evaluate and improve educational interventions for complex problems in a real life environment using a mixed-methods approach [17]. The MRC framework has been developed by the Medical Research Council (MRC) to guide and provide strategies for investigators in recognizing and addressing the challenges of developing and evaluating complex interventions [18]. The three phases of the MRC framework which are used in this study are 1) the development, 2) feasibility and piloting, and 3) evaluation of complex interventions in healthcare.

**Fig. 1:**
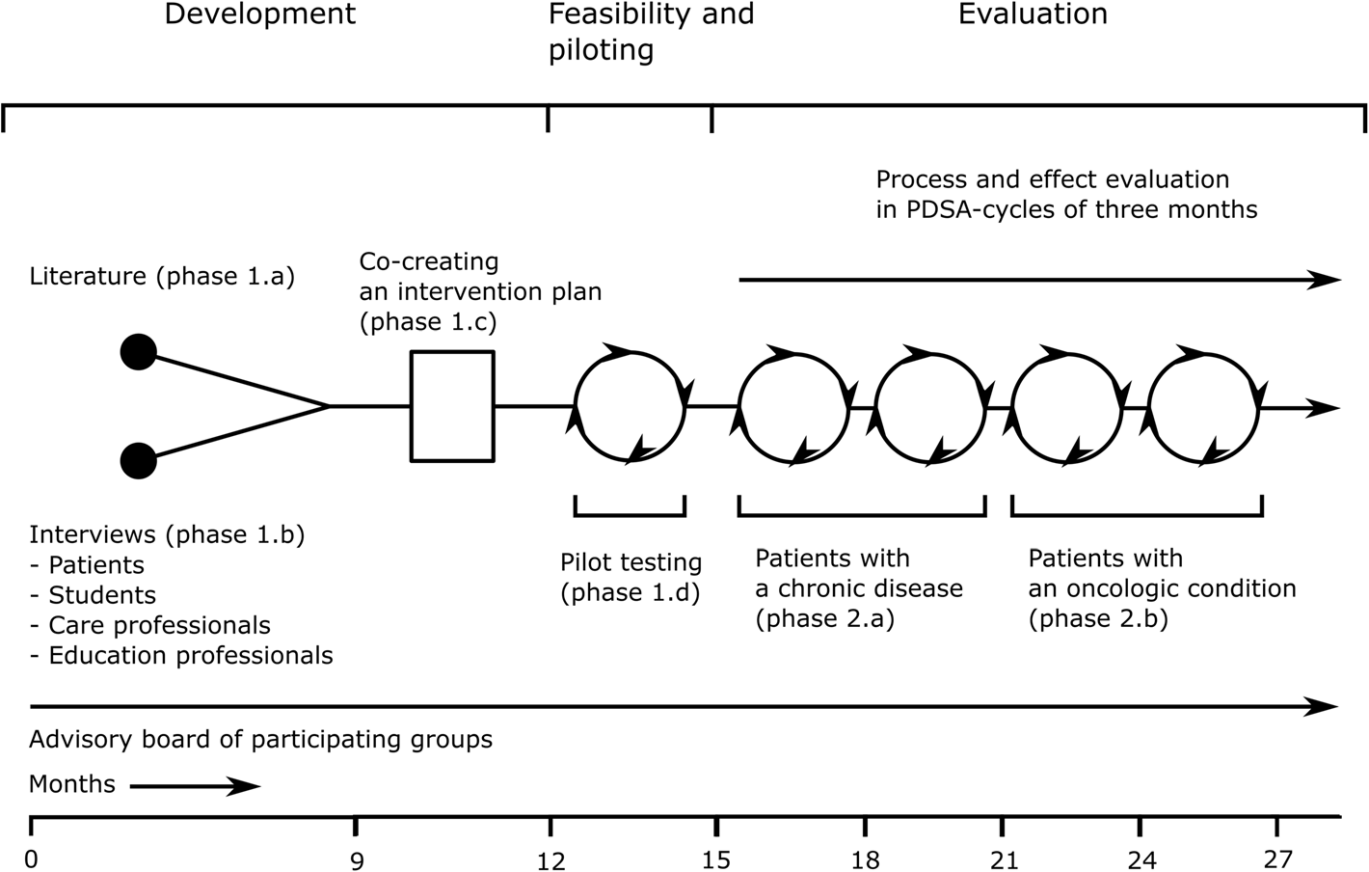
Overview of study protocol. Legend: Overview of the study protocol, which shows the different phases of the design and evaluation study in comparison with the phases of the MRC framework (top line)

In this study, grounding for the design of the training program will be obtained by performing a scoping review of the scientific literature regarding interventions and effects of student-run patient education, and focus group studies regarding the preferences of patients, students, care professionals and education professionals on the structure and content of the training program. Based on the results of the scoping review and focus group studies, an initial intervention plan of the training program will be constructed. The intervention plan will consist of the structure, content and theoretical principles of the program. Elements of the intervention plan will be tested in practice in a small scale pilot (Fig. 1).

Thereafter, the intervention plan of the training program will be evaluated in care practice and medical education using process and effect evaluation in short plan-do-study-act cycles of 3 months to ensure rapid adaptation of the training program to experiences of participants, observations of the training program in practice and effects on participants. In addition, the process and effect evaluation will be used to update theories in the field of patient-centered care and medical education.

Moreover, an advisory board of patients, students, care professionals and education professionals will be shaped at the start of the project to ensure involvement of participating groups and education professionals in all phases of designing and evaluating the training program.

### Research objectives

1. To examine known interventions and effects of student-run patient education by means of a scoping review (phase 1.a).
2. To assess the preferences of patients, students, care professionals and education professionals regarding the structure and content of the training program in focus group studies (phase 1.b).
3. To combine the results of the scoping review and focus group studies into an initial intervention plan of the program (phase 1.c).
4. To pilot-test and evaluate the initial intervention plan with experienced patients, students and care professionals (phase 1.d).
5. To perform process and effect evaluation of the training program in plan-do-study-act cycles as executed with patients with a chronic disease, medical students, nursing students and care professionals (phase 2.a).
6. To perform process and effect evaluation of the training program in plan-do-study-act cycles as executed with patients with an oncologic condition, medical students, nursing students and care professionals (phase 2.b).

### Phase 1.a: Scoping review

#### Method

The scientific literature regarding interventions and outcomes of patient education as performed by undergraduate medical students will be examined. Four databases will be searched (MEDLINE, Embase, ERIC, PsycINFO) for studies reporting patient education as performed by undergraduate medical students.

Studies that 1) involve undergraduate medical students in providing patient education, 2) are aimed at patient education for real patients, and 3) are aimed at patient-centered outcomes of patient education, that is health attitude, self-care, health literacy, treatment compliance, patient empowerment, students’ communication skills, shared decision-making, and relations between (upcoming) care professionals and patients [19, 20], will be included.

Selection and inclusion of studies will be executed by two independent researchers. Differences in judgment will be discussed to reach final agreement. Finally, references of included studies will be searched for other articles meeting the inclusion criteria.

#### Analysis

The quality of included studies will be assessed using the Quality Assessment Tool for Quantitative Studies [21]. Construct validity and inter-rater reliability were tested before [22].

In addition, the patient education intervention method, effects on patients and students, patient education content, patient target group and students’ stage in medical education of the included studies will be characterized using the Kirkpatrick model. The Kirkpatrick model differentiates the extent to which a training program influenced learners on four levels: satisfaction, learning goals, behavior and impact on practice [23].

Finally, an assessment tool will be designed based on the Learning System Transfer Inventory (LTSI). The LTSI is designed for assessing the transfer of training efforts to work practice, and will be applied in this review to assess facilitators and barriers with regard to organizing and implementing practice-based learning in student-run patient education [24].

### Phase 1.b: Focus group studies

#### Method

To include relevant perspectives in designing the training program, nine focus group interviews with patients, students, care professionals and education professionals will be performed (Table 1). Student participants will be recruited from the Radboudumc and the Hogeschool Arnhem en Nijmegen via open invitation. Care professionals of the departments of Rheumatology and Surgery of the Radboudumc will be approached for participation in the focus group interviews with professionals. A topic guide with specific research questions will be used by an experienced moderator to guide the discussion. The topic guide will include questions about the preferences of participants and education professionals regarding patient education in general and the structure and content of the training program. Focus group interviews will be audio recorded and transcribed verbatim.

**Table 1:** Overview of focus group studies

Focus group interviews	Research questions
Patients	
1. Patients with a chronic disease 2. Patient with an oncologic condition 3. Mix of patients with an oncologic condition and patients with a chronic disease	- Why should patient education be performed? - What subjects should patient education address? - What is the vision of patients on the principle of the training program? - How should the training program look like according to patients?
Care professionals	
1. Care professionals of the department of the patients with a chronic disease 2. Care professionals of the department of the patients with an oncologic condition	- What is the current effort regarding patient education at the clinical departments for patients with an oncologic or chronic disease? - What is the vision of care professionals on the principle of the training program? - What subjects should patient education for patients with a chronic or oncologic condition address in the training program? - What subjects should medical education for students address in the training program? - How should the training program look like according to care professionals?
Students	
1. Interprofessional group of medical and nursing students 2. Medical students 3. Nursing students	- What is the vision of students on the principle of the training program? - What subjects should medical education address in the training program? - What subjects should patient education address in the training program? - How should the training program look like according to students?
Education professionals	
1. Medical education professionals of the Radboudumc.	- What is the vision of education professionals on the principle of the training program? - What educational methods can be applied in the training program to provide patient and medical education for patients, students and care professionals?

Legend: Overview of the design of the focus group studies. The first column shows the participants of the focus groups interviews. The second column shows the research questions for the focus group interviews per group (patients, students, care professionals and education professionals)

#### Analysis

Focus group transcripts will be examined using inductive thematic analysis with coding software Atlas.ti to induce semantic themes from the data based on the questions of the topic guide without using a predefined theory or framework. Thematic analysis is a search for themes that emerge as being important to the description of the phenomenon. It involves careful reading and re-reading of the data in order to identify themes, without using a predefined theory or framework in order to capture all relevant information [25]. Two coders will independently code the transcripts and discuss coding and categorization of the transcript to reach agreement.

Resulting themes and categories will be compared between focus groups to analyze different perspectives on the training program. Moreover, differing and overlapping themes and categories between subgroups will be analyzed, for example comparison between the perspectives of patients with a chronic disease and the perspectives of patients with an oncologic condition, and comparison between the perspectives of medical students and the perspectives of nursing students. By comparing between subgroups, we aim to adapt the training program more specifically to the needs and preferences of participants of the training program.

### Phase 1.c: Designing the training program

A concept map will be created based on the scoping review and focus group studies to structure the results of the scoping review and focus group studies. Concept mapping was developed by Joseph Novak and can be used to design complex structures based on knowledge [26]. One researcher (TV) will create a concept map of the results of the review and focus group studies using concept mapping software CmapTools.

Based on the concept map, facilitators and barriers on learning effectiveness of student-run patient education from the scoping review will be compared with the preferences of participating groups and education professionals as obtained in the focus group studies; corresponding items will form the basis for the design process.

Subsequently, the research team (TV, HW, CF, JK) will together assess individual aspects of the concept map and make decisions on the conceptual structure of the program. In the decision process, the preferences of patients, students, care professionals and education professionals, as obtained in the focus group studies, will be used to select items on the structure of the training program which resulted from the scoping review and focus group studies. After that, the conceptual structure will be presented to the advisory board and discussed step-by-step to adapt it to the needs and preferences of participants.

The content of the program will be developed using the Dick and Carey model [27], which describes fundamental steps in the process of designing and evaluating educational interventions. The preferences of patients, students and care professionals as obtained in the focus group studies will be used in the model to build the content of the program.

Finally, the conceptual structure and content of the program will be matched with theories in the field of patient-centered care and learning theories to form the theoretical underpinning for the intervention plan of the program.

### Phase 1.d: Pilot testing the training program

#### Method

To test the initial intervention plan of the training program, a pilot study of the training program will be delivered with expert-by-experience patients who have rheumatoid arthritis for at least more than 10 years (*n* = 6), clinical stage medical students (*n* = 6) and a care professional in the field of rheumatoid arthritis. During the pilot study, learning modules consisting of essential parts of the initial intervention plan of the training program will be provided and evaluated. Learning modules will form the building blocks of the program and consist of educational meetings wherein patients, students and care professionals learn together to reach a specific learning goal of the program.

Observational notes on the modules in practice will be recorded. The notes will be aimed at the organizational process, interactions between participants, integrity and safety aspects, and general remarkable events during execution of the pilot.

In addition, focus group interviews will be held with participants after executing the pilot to assess their experiences regarding the pilot. The topic guides of the focus group interviews will revolve around the organizational process of the program, experiences of participants, interactions between participants, expectations of participants versus reality, and privacy and safety aspects. Three focus group interviews will be held with 1) a mixed group of two patients, two students and one care professional (*n* = 5) to simulate and evaluate the interaction which took place during the pilot by facilitating discussion about the program between patients, students and care professionals, 2) a group of four students (*n* = 4) and 3) a group of four patients (*n* = 4) to address the specific perspectives of participants regarding the structure and content of the pilot. Focus group interviews will be audio recorded and transcribed verbatim.

#### Analysis

Focus group transcripts and observational notes will be examined using inductive thematic analysis with coding software Atlas.ti to induce semantic themes from the data based on the questions of the topic guide without using a predefined theory or framework [25]. Two coders will independently code and categorize focus group transcripts and observational notes and discuss differences in coding and categorization of the transcript to reach agreement.

Next to assessing the overlapping themes and categories between focus groups interviews, comparison will be made between individual focus group interviews to examine the perspectives and experiences of specific participating groups regarding the structure and content of the pilot.

### Phase 2: Evaluating and optimizing the training program

#### Method

**Fig. 2:**
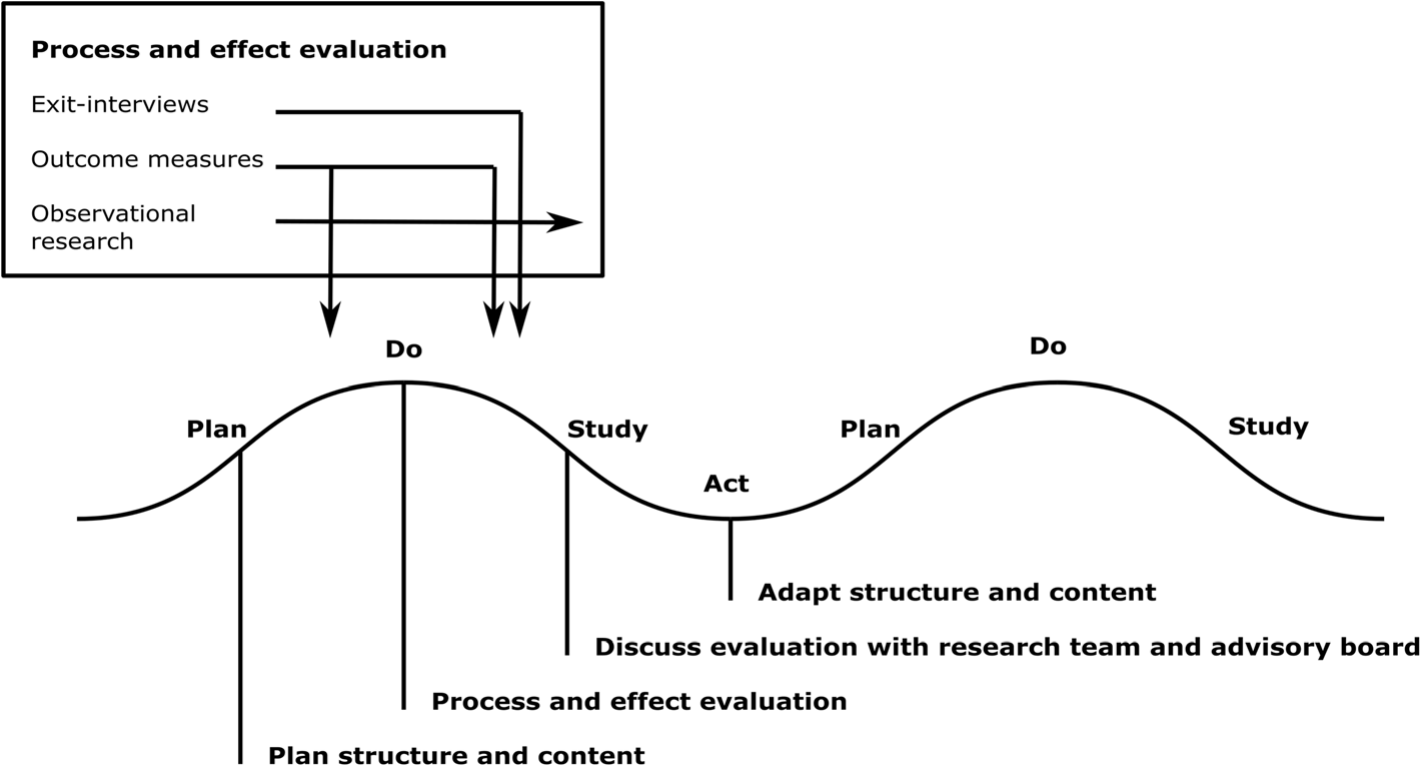
Quality improvement plan in PDSA cycles. Legend: Schematic of the quality improvement plan in the PDSA-cycles of the program. Left-top box shows the methods for process and effect evaluation and time-points in the PDSA-cycle when the research methods are applied. The quality improvement steps in the PDSA-cycles, that is planning the structure and content of the program, performing process and effect evaluation, discussing the evaluation with the research team and advisory board, and adapting the structure and content of the program, are shown at the bottom of the figure

To implement the training program, the plan-do-study-act method (PDSA) will be applied. PDSA is a quality improvement strategy, originating from the engineering industry [28], which enables fast implementation and quality improvement of interventions in healthcare [29, 30]. During a PDSA-cycle, the training program will be planned, performed, evaluated and improved in a period of 3 months during which patients, student and care professionals learn in various learning modules. The process and effects will be evaluated during the PDSA-cycles using various quantitative and qualitative research methods (Fig. 2). The results of the evaluation will be used to improve the design of the program for subsequent PDSA-cycles.

##### Process evaluation

To perform process evaluation, the implementation process, mechanisms of impact and influence of contextual factors on the process will be examined using quantitative and qualitative research methods [31].

Detailed modeling of variations between participants, for example in terms of age, educational level, ethnicity and socio-economic status, will be performed to enhance the process and effect evaluation of the program. In addition, program records on the dose and reach of the program, that is number of sessions, duration of sessions and preparation assignments, participation rate and drop-outs, will be made during execution of the program [31].

A custom-made survey with open-ended and closed-ended questions will be administered to participants after they participated in the program to evaluate participants’ experiences with regard to the training program. Moreover, two qualitative research strategies will be applied during and after the PDSA-cycles to assess the implementation process and fidelity of the program [31]: observational research, and individual exit-interviews or focus group interviews.

Non-participatory observational research will be performed during the training program’s learning modules by taking observational notes on the execution of the program. Observational notes will aim at the organizational process, interactions between participants, integrity and safety aspects, and general remarkable events during the execution of learning modules.

Individual exit-interviews or focus group interviews will be held with participants after each cycle of the training program. The topic guides of individual interviews or focus group interviews will revolve around achieving personal goals during the program, experiences of participants, interactions between participants, expectations of participants versus reality, and privacy and safety aspects. In addition, time will be spend during the exit-interviews or focus group interviews on open comments about the training program.

##### Effect evaluation

It is hypothesized that patients are affected by the program in terms of enhanced disease knowledge, self-efficacy towards care providers, self-efficacy towards chronic diseases, health literacy and patient activation. In addition, it is hypothesized that patients, students and care professionals are affected by the program in terms of a shift of their attitude towards patient-centeredness. Moreover, it is hypothesized that students and care professionals gain more trust in patients whilst participating in the program. Finally, it is hypothesized that interprofessional parts of the program will enhance both medical and nursing students’ attitude towards other care professions and their team skills.

The effects of the training program on patients’ knowledge, attitude, self-efficacy, skills and behavior, and students’ and care professionals’ attitude and skills, will be evaluated by performing before-and-after outcome measures. Based on the above-mentioned hypothesized effects of the training program, reliable and validated measurement instruments are selected for each group and will be used to evaluate the effects of the training program (Table 2).

**Table 2:** Measurement instruments of the effect evaluation

Patients						
Domain	Disease knowledge (kp 2)	Attitude towards patient-centeredness (kp 2)	Health literacy (kp 2)	Self-efficacy in patient-provider relations (kp 2)	Self-efficacy towards chronic diseases (kp 2)	Patient activation (kp 3)
Measurement instrument, Cronbach’s alpha and number of items.	Disease-specific instruments	PPOS [36–38] (α = 0.75 to 0.88) 18 items	HLS-EU-Q47 [39] Domains in healthcare, each 4 items, 16 items in total: Access information (α = 0.68) Understand information (α = 0.73) Appraise information (α = 0.76) Apply information (α = 0.69)	PEPPI-5 [40, 41] (α = 0.92) 5 items	Chronic Disease Self-Efficacy Scale [42, 43] (α = 0.77–0.92) 20 items	PAM-13 [44] (α = 0.91) 13 items
Students						
Domain	Attitude towards patient-centeredness (kp 2)	Trust in patients (kp 2)	Attitude towards other healthcare professions (starting from second cycle, kp 2)	Team skills (starting from second cycle, kp 2)		
Measurement instrument, Cronbach’s alpha and number of items.	PPOS [36–38] (α = 0.75 to 0.88) 18 items	Thom, 2011 [45] (α = 0.93) 18 items	IAQ [46, 47] 27-items (in case of 2 professions)	Team Skills Scale [48, 49] (α = 0.95) 17 items		
Care professionals						
Domain	Attitude towards patient-centeredness (kp 2)	Trust in patients (kp 2)				
Measurement instrument, Cronbach’s alpha and number of items.	PPOS [36–38] (α = 0.75 to 0.88) 18 items	Thom, 2011 [45] (α = 0.93) 18 items				

Legend: Table of outcome measures which are used to evaluate the effects of the program. Rows 2, 5 and 8 show the domains of the effect evaluation and the Kirkpatrick level of the domain (kp) per participating group. Rows 3, 6 and 9 show the applied measurement instruments per domain, the Cronbach’s alpha of the measurement instruments and the number of items used in each instrument

#### Analysis

Process data will be evaluated before the effect evaluation is completed to prevent bias in the interpretation of qualitative evaluations regarding the process. In addition, quantitative and qualitative research methods which are used in process and effect evaluation will build upon another to enhance the evaluation [31]. Theoretical principles, which have been formulated in the intervention plan, will be used to enhance the process and effect evaluation.

Observations will be used to monitor the training program in practice and quickly adapt the training program to participants’ needs and preferences. To do so, observations will be discussed within the research team between learning modules in a PDSA-cycle to examine the implementation process of the training program and adapt the learning modules, if necessary [32].

In addition, observational notes and results of individual exit-interviews or focus group interviews will be assessed in more depth after execution of one PDSA-cycle of the training program. Two coders will independently code and categorize the observational notes and focus group transcripts using thematic analysis to induce semantic themes from the data based on the questions of the topic guide without using a predefined theory or framework [25], and discuss differences in coding and categorization to reach agreement.

Before-and-after outcome measures will be compared to evaluate the effects on patients, students and care professionals using descriptive statistics and multi-level logistic regression analysis. The results of the Patient-Practitioner Orientation Scale will also be compared between participants to compare the effects on attitude towards patient-centeredness between participating groups. In addition, individual questions, items and sub-scales from the measurement instruments will be compared in combination with the process evaluation to assess specific effects of the training program.

All results will be discussed during learning meetings between PDSA-cycles with the research team and the advisory board to examine the implementation process, mechanisms of impact, influence of contextual factors and outcomes of the program, and improve the structure and content of the subsequent PDSA-cycle of the training program (Fig. 2).

Finally, process and effect evaluation will be compared with theories in the field of patient-centered care and medical education to update theoretical principles and enable generalization of the results of our study to other situations.

### Modified control design

**Fig. 3:**
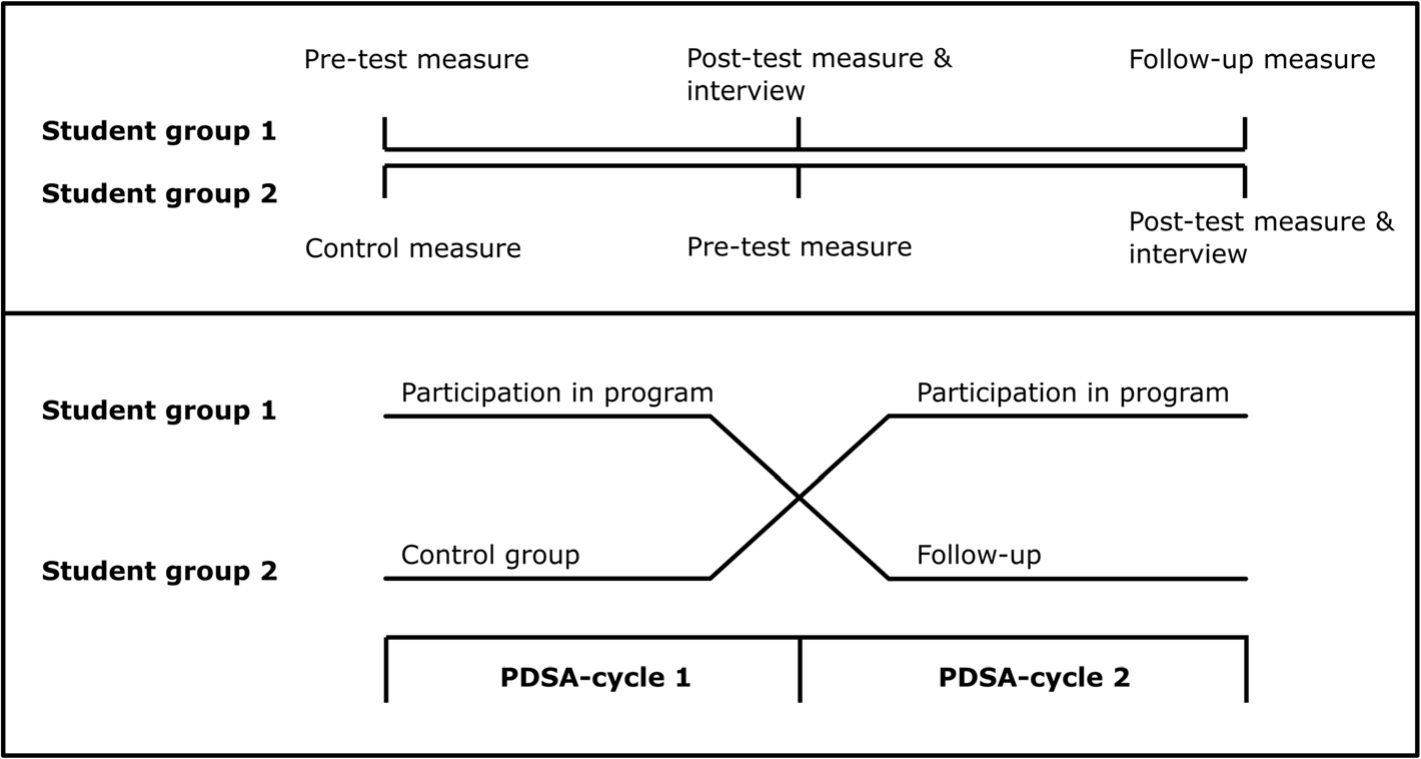
Modified control design for students in the PDSA-cycles. Legend: Schematic of the modified control design as will be used for students in two subsequent PDSA-cycles

A modified control design will be used for two groups of students per two PDSA-cycles to ensure that students who act as control for the intervention group will benefit from participating in the program in a subsequent PDSA-cycle of the program (Fig. 3) [33].

In the modified control design, two student groups simultaneously enter the research program. Student group 1 participates in the program next to the regular curriculum in the first PDSA-cycle and follows only the regular curriculum in the second PDSA-cycle. Student group 2 follows only the regular curriculum in the first PDSA-cycle and participates in the program next to the regular curriculum in the second PDSA-cycle. The process of and effects on student group 1 are evaluated by a pre-test measure with measurement instruments at the start of the PDSA-cycles (T-0), a post-test measure with measurement instruments and exit-interview after following the program (T-1), and a follow-up measure with measurement instruments 3 months after participation (T-2). The process of and effects on student group 2 are evaluated by a control measure with measurement instruments at the start of the PDSA-cycles (T-0), a pre-test measure with measurement instruments before following the program (T-1), and a post-test measure with measurement instruments and exit-interview after following the program (T-2).

Comparison between the outcome measures of both groups at time-points T-0 and T-1 enables controlled evaluation of the effects of the program on student group 1. The outcome measures on student group 1 at time-point T-2 enable uncontrolled follow-up evaluation of the effects of the program on student group 1, 3 months after participation in the program. The outcome measures on student group 2 at time-point T-1 and T-2 enable uncontrolled evaluation of the effects of the program on student group 2.

### Participants

#### Number of participants

For focus group interviews in phase 1.b, we chose to include 4–6 participants per focus group interview since it can be practically challenging to find participants and we want to have a broader range of focus group interviews. For the pilot in phase 1.d, we chose to include six patients, six students and one professional to enable intensive interaction between participants and support the educational nature of the program.

The number of participants in phase 2 will not be calculated using a power calculation due to the explorative nature of the study. We will involve 20–30 participants per cycle to enable interaction between participants in thorough manner and support the educational nature of the program. In addition, we aim to include as many patients as students to facilitate equity between these participating groups. Partially due to the low number of participants and exploratory nature of the protocol, evaluation of the program using descriptive statistics will be aimed at describing the process and effects of the program, instead of explicitly proving the effects of the program.

#### Patients with a chronic disease (phase 1.b, phase 1.d and phase 2.a)

To assess the feasibility of the training program in improving knowledge, attitude, self-efficacy, skills and behavior of chronic patients, patients with rheumatoid arthritis will be included in the first PDSA-cycle of the training program. Patients will participate next to receiving regular care or support. Detailed inclusion criteria for the first cycle and subsequent cycles will be based respectively on the focus group studies, and process and effect evaluations of PDSA-cycles.

After performing process and effect evaluation of the first PDSA-cycle and improving structure and content of the program, a second cycle of the training program will be performed, wherein we plan to include patients with rheumatoid arthritis, diabetes mellitus and hypertension.

#### Patients with an oncologic condition (phase 1.b and phase 2.b)

Oncologic conditions have a different course of disease and influence of the disease on personal life compared to chronic diseases. It is hypothesized that patients with an oncologic condition require a different configuration of the training program. Therefore, after evaluating the training program with chronic patients, the feasibility of the training program in improving knowledge, attitude, self-efficacy, skills and behavior of patients with an oncologic condition, that is colonic cancer and breast cancer, will be evaluated. To do so, patients with an oncologic condition, students and care professionals will participate in subsequent PDSA-cycles of the training program. Patients will participate next to receiving regular care or support.

#### Students (phase 1.b, phase 1.d and phase 2)

To enable students to learn about diseases, the influence of a disease on people’s lives and care processes from the patients’ perspective, medical students and nursing students will participate in the training program.

Medical students will participate in the training program as part of their medical education in the patient-centered medical curriculum of the Radboudumc. Moreover, starting from the second PDSA-cycle, nursing students will be involved in the training program as part of their education at the Hogeschool Arnhem en Nijmegen.

Finally, medical and nursing students will be involved in designing and evaluating the training program by participating in the advisory board and focus group studies. Student internships will be offered to students, in which they contribute to the organization and evaluation of the training program, for example by performing observational research during the PDSA-cycles or contributing to the practical organization of the training programs’ modules.

#### Care professionals (phase 1.b, phase 1.d and phase 2)

Besides patients and students, the training program is hypothesized to affect the attitude towards patient-centeredness of care professionals who participate in the training program, for example by enhancing their knowledge regarding the patients’ perspective. To ensure connection with clinical practice, care professionals who work in the field of chronic diseases and oncologic conditions at the Radboudumc will be involved in the training program by participating in the advisory board, the focus group studies, and the training program itself.

### Ethical issues

To support ethical issues regarding this study protocol, ethical approval of the study protocol has been obtained from the Medical Ethical Committee of the Radboud university medical center for the study parts that include patients. Moreover, ethical approval has been obtained from the Netherlands Association for Medical Education for study parts that include students.

The informed consent procedure will include information briefing prior to inclusion for both patients and students. All participants will have enough time to decide on participation.

All participants will have the possibility to exit the program and/or investigation at any time during execution. Research data obtained until that moment will be deleted.

In addition, interim analyses will be performed after each learning module and PDSA-cycle to decide on continuation of process and effect evaluation by the research team in case of unsafe conditions. Unsafe conditions are mainly possible in psychosocial sense, for example regarding personal information which is communicated during the program or highly emotional issues regarding diseases and treatment. We will monitor execution of the program closely and guide participants during execution by an experienced moderator. Moreover, a safe atmosphere will be created by preparing patients, students and professionals for participation by explaining privacy issues and providing general “rules” of interaction between participants during the program, for example regarding integrity and privacy of communication.

Research data will always be analyzed anonymously. In addition, research data will be stored securely on the server of the department. Personal information will be separated from research data and coded. Only the research team has access to the data.

## Discussion

### Main strengths

**Fig. 4:**
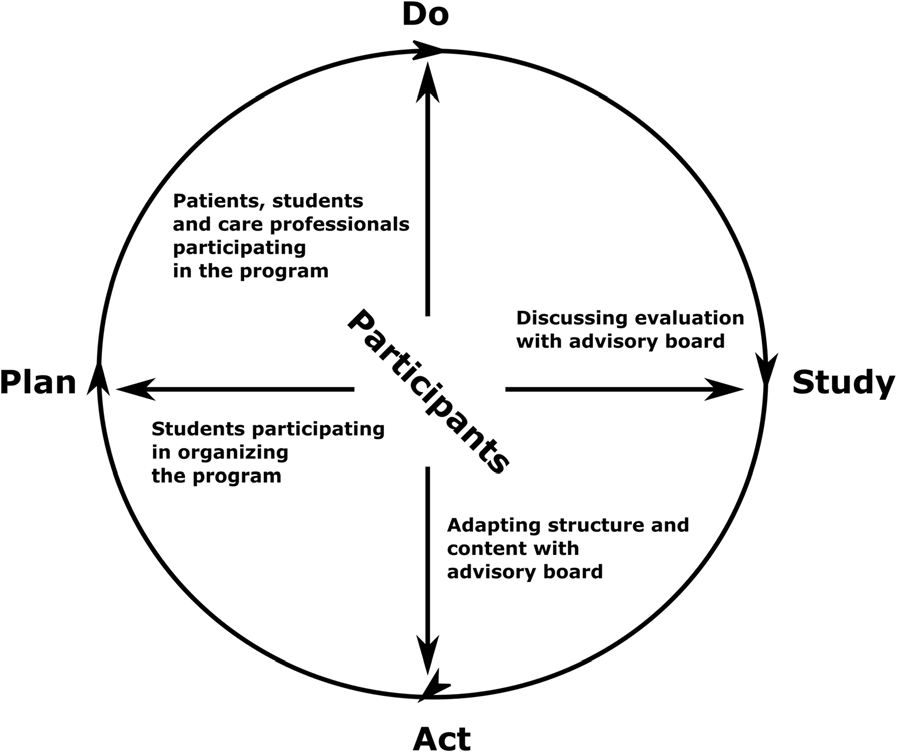
Participatory design of PDSA-cycles. Legend: Schematic of the participatory approach in the PDSA-cycles. The arrows show the involvement of participants in each step of the cycle, that is students participating in organizing the program, patients, students and care professionals participating in the program itself, discussing the evaluation with the advisory board and adapting the structure and content of the program with the advisory board

Our participatory design and evaluation study provides an innovative approach by including various participants in rapid design and evaluation of an educational and therapeutic intervention in care practice and medical education. The participatory approach is expected to enhance the effects of the training program on patients, students and care professionals by meeting participants’ needs and preferences (Table 3, Fig. 4). In addition, by applying short PDSA-cycles of 3 months in evaluating and implementing the training program, the training program can be quickly adapted to participants’ needs and preferences and adjusted to and implemented into care practice and medical education. Moreover, the modified control design in the evaluation phase of our study enables efficient evaluation of the training program by facilitating a control group and follow-up measurement for the effect evaluation on students, whilst simultaneously enabling all students who participate in the study to benefit from participating in the training program.

**Table 3:** Participatory design

The participants of the training program, that is patients, students, care professionals, will contribute to the design and evaluation of the training program in three parts of the project:	
1. *Co-creation:* In the developmental phase of the training program, focus group interviews with participants will be performed to examine the preferences of participating groups regarding the structure and content of the training program.	
2. *Co-production:* An advisory board will be shaped to ensure the input of participants in strategic and operational aspects of designing and evaluating the training program. Moreover, students will contribute to organizing the training program as part of their medical education.	
3. *Co-evaluation.* During the evaluation phase of the project, participants’ perspectives will be assessed in quantitative and qualitative manner to perform program evaluation, which will subsequently be discussed with the advisory board to apply these evaluations in improving the training program (Fig. 4).

### Strengths

Participatory medicine and coproduction of healthcare services are increasingly applied in designing and improving health care. It has been suggested that cooperation between users and providers in medicine will self-evidently improve quality, safety and value of care [34]. In our study, we base the design and evaluation of the program on the needs and preferences of participating groups, and involve them in organizing and improving the program, to ensure that the structure and content of the program reflects their perspectives as much as possible. By doing so, we expect that the actual benefits of the program for participants will be enhanced.

During the process and effect evaluation, a large amount of data will be collected on the implementation process, the mechanisms of impact, the influence of contextual factors and the outcomes of the program. By evaluating the process and effects of the program in broader sense by using various outcome measures, we aim to assess the process and effects of the program in more extent than when only focusing on specific effects. We believe that evaluating only specific effects will not provide in depth insight into the complex learning process that is expected to occur in the program.

By enabling students to meet and interact with patients in an early stage of their educational career, we hypothesize that their attitude towards patient-centeredness and trust in patients will be enhanced (Table 2). In addition, we aim to improve the patient-centeredness skills of medical and nursing students, for example shared decision making skills, communication skills and relations with patients. Enhancing patient-centeredness and patient-centered skills of students in our approach is expected to benefit quality of care and costs of care [2, 35].

In addition, our training program offers patients the opportunity to interact with each other to exchange knowledge and to find support, to obtain personalized information regarding their disease and treatment from students and care professionals, and to improve care services by sharing their experiences with students and care professionals. We expect that the program will improve patients’ knowledge, attitude, self-efficacy, skills and behavior regarding their disease and treatment (Table 2), which can potentially lead to improved quality of care and better health outcomes.

Finally, interaction between care professionals and patients and students in the program is expected to enhance the attitude of care professionals with regard to patient-centeredness (Table 2). Although care professionals have opportunities to learn about the patients’ perspective in regular care, the learning modules of the training program are expected to provide more time and depth with regard to obtaining knowledge and experience about the patients’ perspective. We aim to further investigate the effects of the program on quality and costs of care in future research.

### Challenges

Our study protocol is based on the concept of co-production. We will involve patients, students and care professionals in all phases of designing and evaluating the training program. However, co-production in its purest form would also include patients, students and care professionals executing or analyzing for example focus group interviews, observational notes, and/or results of measurement instruments. Since it is a practical challenge to involve all target groups in all phases of executing this protocol, we incorporated specific measures to involve participating groups as much as possible in the design and evaluation of the program, for example by shaping an advisory board of participating groups to assess results of evaluations, and enabling students to organize the program. In addition, since this study protocol is aimed at designing and evaluating the training program in its earliest phase, we expect to provide more intensive and purer co-production at a later stage of implementing the program, for example by involving former participants in producing, implementing and evaluating the program.

Personalizing the training program to the individual needs and preferences of each participant would require and intensive amount of time, money and effort. In addition, knowledge and skills differ between patients and between (future) care professionals, and it is expected to be a challenge to fit the training program to the capacities of all patients, students and care professionals. We will differentiate between participant subgroups in designing and evaluating the program to fit the training program to the needs, preferences and capacities of individual participants as much as possible. In addition, we will assess if the program fitted participants’ personal needs, preferences and capacities during program evaluation, and use these experiences to improve the next cycle of the program.

In addition, prioritizing the experiences and opinions of participants as presented during the program evaluation will be essential to be able to improve the training program in the PDSA-cycles. To be able to apply the preferences of participants in improving the training program, we will discuss and prioritize the evaluations with the advisory board.

Since the program is evaluated in one setting, the transferability of the results of our study towards other settings might be limited. To address the transferability, we will use theoretical principles to ground the intervention plan and explain the results of process and effect evaluation. In addition, we will describe the participants, the context of the learning environment of the program and our analytic approach in depth to enable comparison of our results to other situations [17].

Design-based research can be risky due to uncertainties in participant behavior and circumstances in the learning environment, and complicated in the sense of combining theory and practice while involving various stakeholders during all steps of designing and evaluating an intervention [17]. To address the uncertainties during the evaluation phase, we will enable fast adaptation of the program’s structure, content and execution to the results of process and effect evaluation between PDSA-cycles. In addition, to address the complexity of organizing and evaluating the program, we will start with evaluating the program at a small scale by involving a limited amount of participants and using a simple set-up of the training program. At a later stage, we aim to expand to more PDSA-cycles of the program for different patient groups, evaluate more complex designs of the training program (for example by involving more students of other disciplines or combining different patient groups in one program) and provide the training program at various locations (for example by providing the program in the community or online). Moreover, once evaluated at a small scale, we aim to include the program in the regular curriculum and form an organizing board of patients and students to enhance our participatory approach.

Finally, medical and nursing students are commonly prepared for ethical and moral aspects of care practice at the end of their educational career. However, since undergraduate medical and nursing students are not yet fully prepared and equipped for these aspects of care practice, it is essential to monitor the integrity and safety of the training program. In addition, discussing severe diseases and the influence of a severe disease on the lives of patients between patients, students and care professionals can have psychological impact on both patients and students. To ensure the integrity and safety of both patients and students, we will pay attention to the psychological and social aspects of participating in the training program during observational research and program evaluation. Moreover, to ensure an ethically safe design of the training program, ethical approval will be requested for both patient and student participation in the project from respectively the ethical committee of the Radboudumc and the ethical committee of the Dutch Association for Medical Education.

### Summary

To summarize, in our participatory design and evaluation study, an innovative patient-centered and interprofessional training program is co-created together with patients, students and care professionals. By embedding the perspectives of participating groups in all phases of designing and evaluating the training program, we aim to meet the needs and preferences of patients, students and care professionals and enhance the effectiveness of the training program. Moreover, by using fast PDSA-cycles and a modified control design in evaluating the training program, we aim to adjust the training program rapidly to process and effects evaluation, and efficiently evaluate and optimize the program in care practice and medical education.

## Abbreviations

LTSI: Learning transfer system inventory
MRC: Medical Research Council
PDSA: Plan-do-study-act
